# Interaction between Axons and Specific Populations of Surrounding Cells Is Indispensable for Collateral Formation in the Mammillary System

**DOI:** 10.1371/journal.pone.0020315

**Published:** 2011-05-20

**Authors:** Nora-Emöke Szabó, Tianyu Zhao, Murat Çankaya, Anastassia Stoykova, Xunlei Zhou, Gonzalo Alvarez-Bolado

**Affiliations:** 1 Brain Development Group, Max Planck Institute of Biophysical Chemistry, Göttingen, Germany; 2 Department of Biology, Faculty of Sciences and Art, Erzincan University, Erzincan, Turkey; 3 Department of Molecular Cell Biology, Max Planck Institute of Biophysical Chemistry, Göttingen, Germany; Tokyo Medical and Dental University, Japan

## Abstract

**Background:**

An essential phenomenon during brain development is the extension of long collateral branches by axons. How the local cellular environment contributes to the initial sprouting of these branches in specific points of an axonal shaft remains unclear.

**Methodology/Principal Findings:**

The principal mammillary tract (pm) is a landmark axonal bundle connecting ventral diencephalon to brainstem (through the mammillotegmental tract, mtg). Late in development, the axons of the principal mammillary tract sprout collateral branches at a very specific point forming a large bundle whose target is the thalamus. Inspection of this model showed a number of distinct, identified cell populations originated in the dorsal and the ventral diencephalon and migrating during development to arrange themselves into several discrete groups around the branching point. Further analysis of this system in several mouse lines carrying mutant alleles of genes expressed in defined subpopulations (including *Pax6*, *Foxb1, Lrp6* and *Gbx2*) together with the use of an unambiguous genetic marker of mammillary axons revealed: 1) a specific group of *Pax6*-expressing cells in close apposition with the prospective branching point is indispensable to elicit axonal branching in this system; and 2) cooperation of transcription factors *Foxb1* and *Pax6* to differentially regulate navigation and fasciculation of distinct branches of the principal mammillary tract.

**Conclusions/Significance:**

Our results define for the first time a model system where interaction of the axonal shaft with a specific group of surrounding cells is essential to promote branching. Additionally, we provide insight on the cooperative transcriptional regulation necessary to promote and organize an intricate axonal tree.

## Introduction

**Figure 1:**
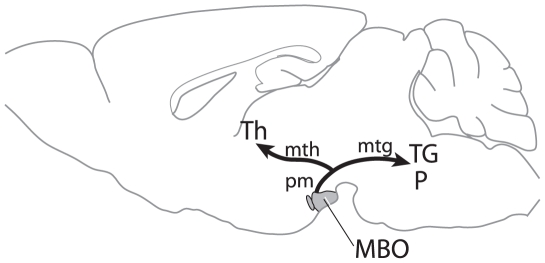
The mammillary body and its efferents as classically described. Diagram of MBO efferent connections to diencephalon and brainstem. P, pons; TG, tegmentum. Other abbreviations: see text.

Outgrowing axons commonly branch immediately proximal to the growth cone sending offshoots to nearby targets [1]. However, stereotyped (i.e. identical in all individuals) axonal collaterals form through sprouting and branching at the axonal shaft away from the growth cone [2], [3]. Although it remains unclear how the precise branching points are initiated, it has been suggested that cells in close apposition to the axon could contribute to branching [4]. Here we use the development of the pm (Fig. 1) and its surrounding cells as a model to study the possible interaction between local environment and axonal collaterals. The mammillary body (MBO) is a nuclear complex in the postero-ventral diencephalon with defined functions in learning and memory [5]. The MBO generates the pm which is continued by the mtg (Fig. 1). The mammillothalamic tract (mth) is a large, stereotyped collateral of the pm connecting MBO with thalamus (Th in Fig. 1) [6]. The mammillotectal tract (mtc) connects MBO to the tectum [7], [8].

We approached this model through analysis of its development in wild type and in several mouse lines carrying null phenotypes for genes expressed in identified cellular subpopulations surrounding the branching point. We also made use of the *Foxb1-tauLacZ* allele, an unambiguous genetic marker of mammillary axons.

Our results show that the future branching point in the pm is marked by a complex arrrangement of specific cells including a unique cell group formed by at least two distinct, specific subpopulations originated, respectively, in the ventral and in the dorsal diencephalon. We found evidence strongly supporting that interaction between the axonal shaft and specific populations of surrounding cells is indispensable for collateral branching. Additionally, we show that *Foxb1* cooperates with *Pax6* to differentially regulate navigation of mammillary axonal bundles targeting the tectum and tegmentum, probably through control of fasciculation.

## Materials and Methods

### Mouse lines

Animals were handled in ways that minimize pain and discomfort, in agreement with the European Communities Council Directive (86/609/EEC). To obtain embryos, timed-pregnant females of the appropriate crossings were killed by cervical dislocation.

#### *Foxb1-tau-lacZ*

This mouse mutant line [7] carries axonal marker tau-lacZ [9] as a reporter of *Foxb1* expression. *Foxb1* heterozygotes show normal phenotype [7], [10]–[12] and no homozygotes were used in this study. Since *Foxb1* is specifically expressed in the MBO including the dorsal premammillary nucleus [7], [13] expression of beta-galactosidase in heterozygotes provided us with a clear-cut genetic marker of this nuclear complex and its axonal projections.

#### *Foxb1::Cre*

This line carries the Cre recombinase under the control of *Foxb1* regulatory sequences (knockin-knockout) [14]. Upon crossing with reporter line ROSA26R [15], it reveals the *Foxb1* cell lineage [16].

#### *Pax6-Small eye (Sey)*

A spontaneous null mutant allele of *Pax6* [17], [18].

#### *Pax6::lacZ*

This targeted null allele of *Pax6* expresses beta-galactosidase as expression reporter [19].

#### *Lrp6* mouse mutant line

Courtesy of Dr. Kenji Imai (Helmholtz Center Munich, Germany) [20].

#### *Gbx2* mouse mutant line

Courtesy of Drs. Gail Martin (University of California San Francisco) and Alex Joyner (Sloan-Kettering Cancer Center, New York).

### Immunohistochemistry

Embryos of the appropriate ages were obtained and fixed by immersion in paraformaldehyde 4% in phosphate buffer saline (PBS). Paraffin sections (15 micrometer) of mouse brains were dewaxed, preincubated in PBT/10% fetal calf serum and incubated overnight (4°C) in rabbit anti-beta-galactosidase antibody (Molecular Probes-Invitrogen Cat. Nr. A11132), or chicken anti-beta-galactosidase antibody (1∶500) (Abcam Cat. Nr. 9361) and/or mouse monoclonal anti-Pax6 antibody (1:50) (Developmental Studies Hybridoma Bank). Either fluorescent secondary antibodies (Alexa 488 and Alexa 594, Invitrogen), or biotinylated antibodies (Vector Laboratories, Cat. Nrs. BA-9010, BA-9200 or BA-1000) followed by Streptavidin-POD (GE Healthcare, RPN 1231V) and diaminobenzidine (Sigma-Aldrich, D3939) were used for visualization.

### *In situ* hybridization

Was performed on cryostat sections of fresh-frozen embryo brains according to current protocols [16], [21], [22].

### Counting axons on histological sections

Immunodetection of beta-galactosidase was performed on sagittal paraffin sections of three E16.5 brains per genotype. E16.5 was chosen since at this age there is no mth yet in normal animals (see Results section). Three sections were counted per side of the brain, and the right and left sides of the brain were considered separately. The immuno-labeled axons coming out of the dorsal side of the pm were scored as belonging to one of two groups— the ones oriented rostro-dorsally (the “problem axons”, see Results section) from the ones oriented caudo-dorsally (mtc). Statistic analysis was performed with Prism software (GraphPad, La Jolla, California).

### Axonal tracing with DiI

The lipophilic carbocyanine dye DiI (Invitrogen, Darmstadt, Germany) was dissolved (25%) in dimethylformamide and a very small amount of the solution (it is not possible to know exactly how much) was injected in paraformaldehyde-fixed brains with a glass capillary. The brains were left at 37°C protected from the light for several days, then embedded in 4% agarose, cut with a vibrating microtome and analyzed and photographed in a fluorescence microscope with a rhodamine filter.

### Microscopy

Nikon A1 confocal (Nikon Engineering, Yokohama, Japan), Leica DMR and MZ APO microscopes (Leica Mikrosysteme, Wetzlar, Germany), Olympus DP50 cameras (Olympus, Tokyo, Japan) and Cell-F 2.6 software (Olympus Soft Imaging Solutions GmbH, Münster, Germany) were used for analysis and photography. Image contrast was enhanced by applying Photoshop 7.0 software tools (Adobe Systems Inc., San José, California) to one whole image file at a time. IMARIS software (Bitplane, Zürich) was used for reconstructions of DiI-labeled axons.

## Results

### Arrangement of specific cell groups at the pm branching point

**Figure 2:**
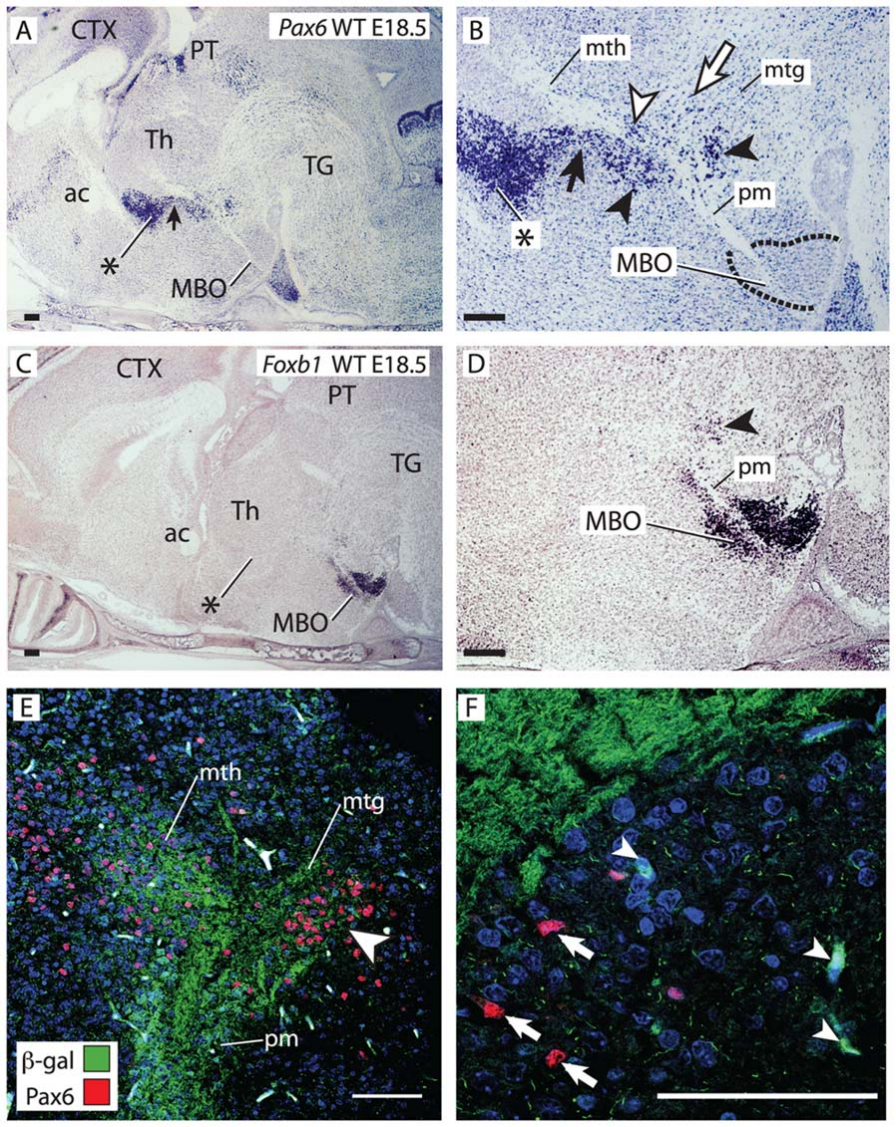
A complex and specific cell aggregate around the bifurcation point. A–D) In situ hybridization for *Pax6* (A, B) and *Foxb1* (C, D) on sagittal sections of wild type E18.5 brains (rostral to the left). (B) and (D) show high magnification details of (A) and (C). Black arrowheads, specific cell groups around the branching point. ac, anterior commissure; CTX, cortex; PT, pretectum. B) *Pax6*-expressing cells are also found forming a trail under the mth (black arrow) continuous with the PTh/VTh (asterisk), between the mth axons (white arrowhead), and in the area between mth and mtg (white arrow). E, F) Confocal pictures of antibody detection of Pax6 (red cell nuclei) and beta-galactosidase (green cell bodies; proxy for *Foxb1* expression) on a sagittal section of an E18.5 *Foxb1-tau-lacZ* heterozygous brain. Blue labeling, DAPI nuclear staining. E) Double labeling of the branching point shows a compact group of Pax6- and Foxb1-positive cells (arrowhead). F) *Foxb1*-positive (arrowheads, green cell bodies) and *Pax6*-positive (arrows, red nuclei) cells are distinct from each other. Asterisk in A, B, C: PTh/VTh. Scale bars 100 micrometers.

The pm branching point finds itself in the posterior hypothalamus (ventral diencephalon), dorsal to the mammillary body, and approximately in register with the boundary between two dorsal diencephalic subdivisions classically named dorsal and ventral thalamus. Based on recent advances in our understanding of diencephalic development a new terminology is being introduced (see for instance [23]–[25]) in which the names prethalamus (formerly known as ventral thalamus) and thalamus (formerly known as dorsal thalamus) are preferred. In order to avoid confusion, we will call these two structures prethalamus/ventral thalamus (PTh/VTh, labeled in the Figures with an asterisk) and thalamus (Th). Transcription factor gene *Pax6* is a marker of PTh/VTh [26], [27] and we used it as the basis of our analysis. We found a trail of *Pax6*-positive cells joining the most ventral end of the PTh/VTh to the branching point (black arrow in Fig. 2A, B). Closer examination (Fig. 2B) revealed an intriguing and complex distribution of *Pax6*-expressing cells around the mammillary axonal tree. The *Pax6*-expressing trail of cells was in contact with the mth and ended in a group of cells closely apposed to the branching point (black arrowheads in Fig. 2B). *Pax6*-positive cells were also present between the mth axons (white arrowhead in Fig. 2B) lending the first stretch of this tract its characteristic reticulate appearance [7], [28]. Finally, numerous *Pax6*-positive cells were found scattered in the area defined by the mtg and the mth (white arrow in Fig. 2B). Transcription factor *Foxb1* is a specific marker of the MBO (Fig. 2C, D) [7], [29]. We detected a group of *Foxb1*-expressing cells apposed to the caudal side of the branching point (arrowhead in Fig. 2D). To elucidate the relation between the *Pax6*-expressing and the *Foxb1*-expressing cells around the pm branching point, we performed double immuno-staining for beta-galactosidase and Pax6 on E18.5 *Foxb1::tau-lacZ* heterozygous brains [7] (beta-galactosidase detection indicates *Foxb1* expression and the tau-beta-galactosidase fusion protein is localized to the corresponding axons) (Fig. 2E). The results showed a group of Pax6-positive cells and Foxb1-positive cells (arrowhead in Fig. 2E) in the caudal side of the branching point. Closer observation at higher magnification (Fig. 2F) revealed that marker expression was mutually exclusive—no green labeled cell somata (Foxb1-positive) (white arrowheads in Fig. 2F) had red nuclei (Pax6-positive) (white arrows in Fig. 2F).

### The *Foxb1*-expressing cells originate in the MBO

**Figure 3:**
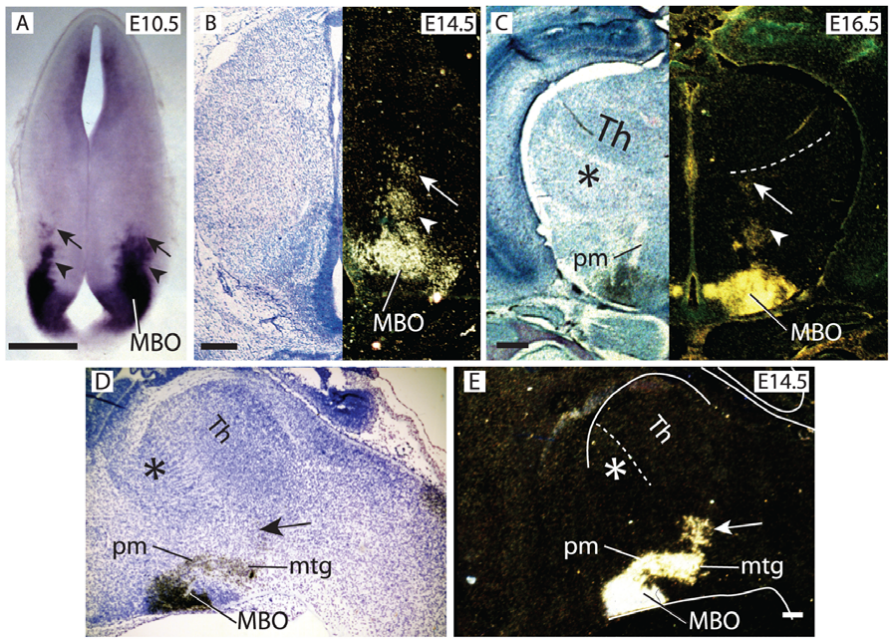
*Foxb1*-expressing cells migrate from the MBO along the pm. A) *Foxb1* expression on a transverse section of a wild type E10.5 brain. Arrowheads, column of *Foxb1*-expressing cells originated in the MBO and migrating dorsally, preceded by a pioneer group (arrow). B, C) *Foxb1* expression on transverse sections of wild type E14.5 (B) and E16.5 (C) brains. Left side shows Nissl counterstaining, right side shows dark field. Dotted line in C, E, external medullary lamina (zona limitans). D, E) *Foxb1* expression in a sagittal section of an E14.5 wild type brain. (D) shows Nissl counterstaining, (E) shows dark field. Arrow, pioneer group of *Foxb1*-expressing cells. Asterisk in C, D: PTh/VTh. Scale bars A, B, C: 50 micrometers; E: 25 micrometers.

Since *Foxb1*-positive and *Pax6*-positive cells are distinct populations, we asked if they have different origins. Detection of *Foxb1* expression on wild type embryonic brains at E10.5 (Fig. 3A) revealed strong expression in the MBO [29] as well as in a “column” spreading dorsally from this nucleus (arrowheads in Fig. 3A) and in a more dorsal, looser group of cells (arrows in Fig. 3A). This column of *Foxb1*-expressing cells expanded dorsally through E14.5 (Fig. 3B) and E16.5 (Fig. 3C), finally reaching the boundary between thalamus and PTh/VTh in the dorsal diencephalon (dotted line in Fig. 3C). The *Foxb1*-positive cell column seemed to be apposed to the lateral side of the pm axons (Fig. 3B, C).

Sagittal sections (Fig. 3D, E) confirmed that the labeled cells form a numerous group along the pm and mtg, and there is a looser group more dorsally positioned at the future branching point (arrow in Fig. 3D, E).

### The *Pax6*-expressing cells originate in the PTh/VTh and are missing in *Pax6*-deficient brains

**Figure 4:**
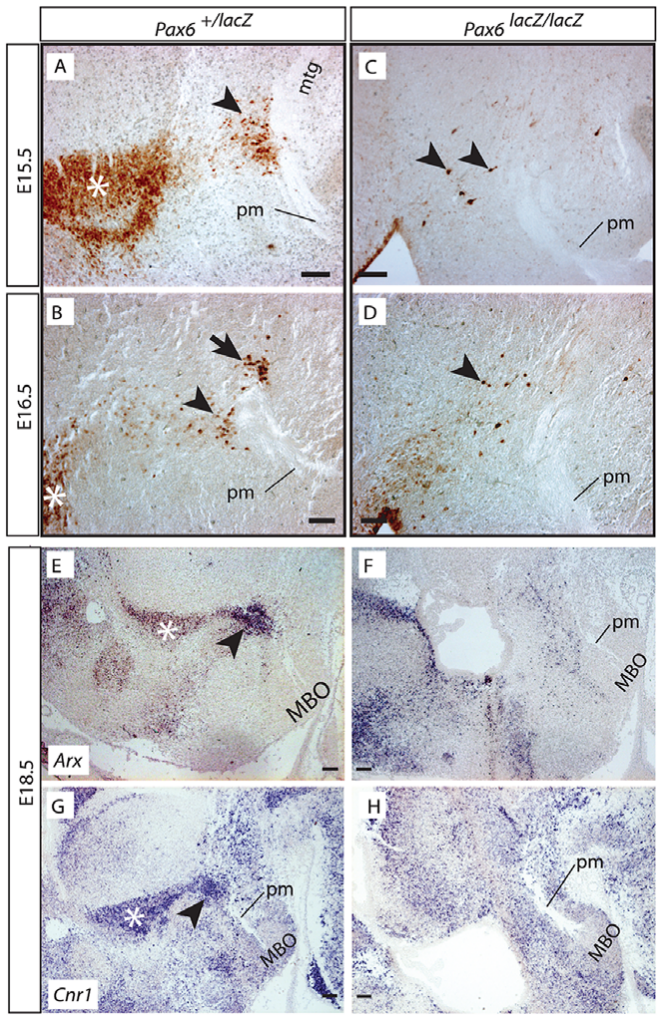
*Pax6*-expressing cells are continuous with the PTh/VTh and are missing in the *Pax6* mutant. A–D) Beta-galactosidase antibody detection on sagittal sections, rostral to the left. Ages and genotypes as indicated. A trail of *Pax6*-expressing cells (arrowheads in A, B) from the PTh/VTh lands on the pm branching point at E15.5 (arrowhead in A). At E16.5 there is a second labeled cell group on the caudal side (arrow in B). In the *Pax6* mutant these cells (arrowheads in C, D) are very scarce and do not contact the pm. E–H) In situ hybridization detection of PTh/VTh markers on sagittal sections. Both *Arx* (E) and *Cnr1* (G) expression label the branching point cells continuous with the PTh/VTh (arrowheads in E, G). Both markers are absent in the *Pax6*-deficient diencephalon (F, H). Asterisk in A, B, E, G: PTh/VTh. Scale bars 100 micrometers.

To elucidate the origin of the *Pax6*-expressing cells we used the *Pax6-lacZ* mouse line, which carries a null mutation of *Pax6* followed by *lacZ* as reporter [19] (see also Table 1). In E15.5 heterozygotes, a trail of beta-galactosidase-positive cells can be followed from the PTh/VTh to a specific point of the rostral side of the pm, where they aggregate (arrowhead in Fig. 4A). By E16.5, in heterozygous brains the trail of beta-galactosidase-positive cells connecting PTh/VTh and pm is still evident (Fig. 4B). In addition to the labeled cell group on the rostral side of the pm (arrowhead in Fig. 4B), a second group is forming on the caudal side (arrow in Fig. 4B). Because of the close proximity between the cells and the branching point, we hypothesized that they play a role in the branching process. Since mice deficient in *Pax6* lack a PTh/VTh [27], we first asked if the branching point cells are also absent in these mutants. Homozygous brains at E15.5 showed only very few reporter-expressing cells in this region (arrowheads in Fig. 4C), and none of them reached the pm. Homozygotes at E16.5 showed again few labeled cells and none of them was situated next to the pm (arrowhead in Fig. 4D). Other PTh/VTh marker gene, *Arx* [30] (Fig. 4E) also labels the trail of cells between PTh/VTh and pm as well as a cell group around the pm branching point. Examination of the expression pattern database www.genepaint.org (in the public domain) in search for other markers of this region suggested that the cannabinoid receptor *Cnr1* [31] could be a good candidate. Our in situs confirmed this, since *Cnr1* is expressed like *Arx* and *Pax6* in this region (Fig. 4G). Both *Arx* and *Cnr1* confirmed the lack of PTh/VTh cells around the pm in the mutant (Fig. 4F, H).

**Table 1:** *Foxb1* and *Pax6*: Mutants and Phenotypes.

Mutant	Null mutant for:	Reporter Gene	Problem axons	mth	pm	*Foxb1* BPC^1^	*Pax6* BPC^1^
*Pax6 ^+/lacZ^*		b-gal^2^ in PTh/VTh^4^ and *Pax6*-BPC^1^	very few	yes	---	---	yes
*Pax6 ^lacZ/lacZ^*	*Pax6*	b-gal^2^ (in the sparse remnants of PTh/VTh^4^)	abundant	no	---	---	no
*Pax6^Sey/Sey^*	*Pax6*	none	abundant (DiI)	no (DiI)	loose (DiI)	---	---
*Foxb1-tau-lacZ* ^+/−^		b-gal^2^ in mam^3^ axons and *Foxb1*-BPC^1^	very few	yes	tightly bound	compact	yes
*Foxb1-tau-lacZ ^+/−^ Pax6 ^Sey/Sey^*	*Pax6*	b-gal^2^ in mam^3^ axons and *Foxb1*-BPC^1^	abundant	no	loose	loose	no
*Foxb1-tau-lacZ ^−/−^ Pax6 ^Sey/Sey^*	*Foxb1 Pax6*	b-gal^2^ in mam^3^ axons and *Foxb1*-BPC^1^	very abundant	no	very loose	very loose	no
*Foxb1-tau-lacZ ^−/−^*	*Foxb1*	b-gal^2^ in mam^3^ axons and *Foxb1*-BPC^1^	very few	yes	tightly bound	compact	yes

The mutants are listed in the order they appear in the Results section. Two different *Pax6* mutants, with and without reporter were used. The *Pax6*-driven reporter (*Pax6-lacZ*) labels the PTh/VTh and *Pax6*-BPC, but not the mammillary body, axons or *Foxb1*-BPC. The *Pax6 Sey* mutant carries no reporter and its phenotype is analyzed by DiI axonal tracing. The *Foxb1-tau-lacZ* mouse carries a *Foxb1*-driven reporter labeling the mammillary body and axons and the *Foxb1*-BPC. The pm and *Foxb1* BPC have not been examined in the *Pax6*-*lacZ* mutant because they express neither *Pax6* nor the *Pax6*-driven *lacZ* reporter.

1 BPC: Branching Point Cells;

2 b-gal: beta-galactosidase;

3 mam: mammillary;

4 PTh/VTh: Prethalamus/Ventral thalamus.

We concluded that the branching point cells are an extension of the PTh/VTh and that, like the rest of the PTh/VTh, they are absent in the *Pax6* mutant.

### Mammillary axons growing towards the thalamus in the *Pax6* mutant

**Figure 5:**
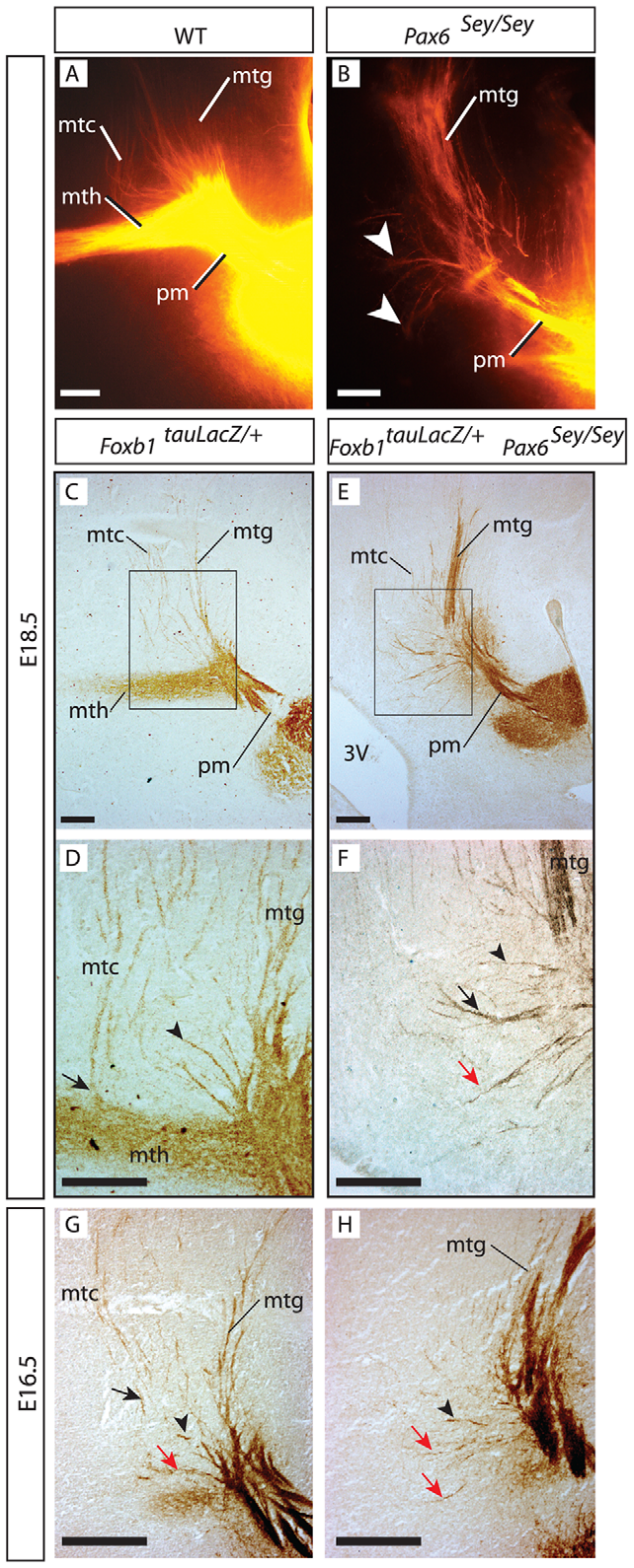
Thalamus-oriented axons in the *Pax6*-deficient diencephalon. A, B) DiI tracing on sagittal sections of E18.5 wild type (A) and *Pax6* homozygous (B) embryos. In the mutant, a few short axons (arrowheads in B) can be seen in place of a mth. C–H) Antibody detection of beta-galactosidase on sagittal sections, ages and genotypes as indicated. D, F are high magnification details the frames in C, E. C, D) Some mtc axons navigate directly towards the tectum (arrowhead in D) and others course towards the thalamus, then sharply change direction (arrow in D). E, F) In the *Pax6* mutant, similar mtc axons can be seen changing course towards the tectum (black arrow in F), others grow straight dorsally (arrowhead in F) and finally others grow rostrally in the direction of the thalamus (red arrow in F). 3V, third ventricle. G, H) At E16.5, before the mth appears, there are mtc axons in *Pax6* wild type (G) and mutant (H) (arrows and arrowheads as in F). Scale bars 100 micrometers.

We then analyzed the mammillary axonal tree in wild type and in the *Pax6* mutant by injecting DiI tracer into the MBO (Fig. 5A, B). In the wild type, the mth, mtg and mtc were easy to recognize (Fig. 5A). In the mutant diencephalon, the mth was absent. Instead, there was a number of axons apparently originated in the branching point and sometimes oriented towards the thalamus (arrowheads in Fig. 5B) which are however less in number and of shorter length than the axons of the wild type mth. They also lack the characteristic morphology of the early mth axons (thin, beaded axons weaving their way around local cell bodies that leave “holes” in an otherwise compact bundle) [7], [28]. We termed them “problem axons” and set out to investigate their origin.

### The problem axons develop earlier than the mth

To label the mammillary axons unambiguously, we crossed the *Pax6-*deficient *Small eye* (*Sey*) mutant, carrying no reporter gene, [17], [27] with the *Foxb1-tau-lacZ* transgenic line and used anti-beta-galactosidase antibody to compare the axons of *Foxb1-tau-lacZ* heterozygous embryos (normal embryos) (Fig. 5C, D, G) (see also Table 1) to those of double mutant embryos (*Foxb1-tau-lacZ* heterozygous/*Sey* homozygous) (Fig. 5E, F, H).

**Figure 6:**
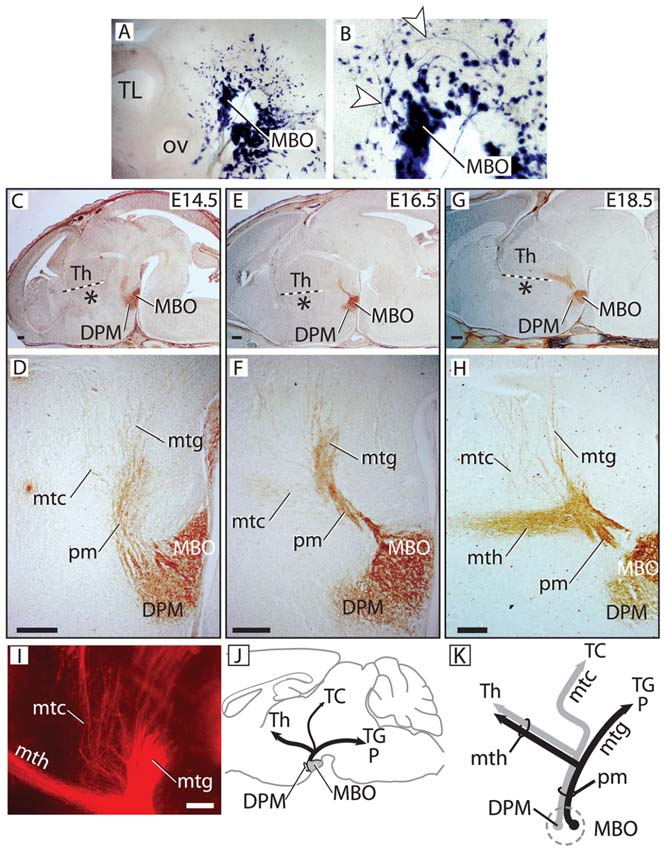
Stepwise development of the three components of the mammillary axonal tree. A, B) Beta-galactosidase activity detection in the flat-mounted right side of an E10.5 *Foxb1::Cre x ROSA26R* heterozygous brain showing the first axons (arrwoheads in B) from the MBO navigating towards the tegmentum. Rostral to the left. (B) shows a high magnification detail of (A). ov, optic vesicle; TL, telencephalon. C–H) Antibody detection of beta-galactosidase on sagittal sections of *Foxb1-tau-lacZ* heterozygous brains. D, F, H show high magnification details of C, E, G, respectively. The dotted line in C, E, G marks the boundary between PTh/VTh and Th. C, D) The first mtc axons detach from the pm at E14.5. E, F) At E16.5 the pm acquires a pronounced bend marking the origin of the mtg. G, H) The mth appears at E18.5, branching from the bend in the pm observed at E16.5. I) DiI tracing shows the components of the mammillary axonal tree at E18.5. J) Diagram of MBO efferent connections to diencephalon and brainstem. K) Diagram of mammillary efferent axons. Grey, dorsal premammillary axons. Black, axons from the MBO proper. Asterisk in C, E, G: PTh/VTh. Scale bars: C, E, 25 micrometers; G, 50 micrometers; D, F, H, I, 100 micrometers.

Analysis of *Foxb1-tau-lacZ* heterozygotes at E18.5 showed that the mth and mtg separated from each other at right angles leaving a broad area between them occupied by mtc axons not forming an obvious bundle (Fig. 5C, D). Some mtc axons follow the mth for a short stretch to separate later at right angles, while others spread from the beginning over a wide area (Fig. 5C and see Fig. 6I below). Some of these loose axons spread over the “decision area” between mth and mtg, were oriented caudo-dorsally towards the tectum (Fig. 5D, arrowhead) while others followed originally a dorsal trajectory first, before turning sharply into the caudal direction (Fig. 5D, arrow). In the *Pax6* mutant at E18.5 (Fig. 5E, F), some of the problem axons followed a caudal path similar to some of the non-bundled axons found in the wild type (Fig. 5F, black arrow and arrowhead). There was however a number of short axons extended in a dorsal and rostral direction towards the thalamus (Fig. 5F, red arrow). We asked if these short, thalamus-oriented axons were also present in the wild type, but hidden by the mth. To solve this question we analyzed mutants at an earlier age, E16.5, when there is no mth yet in the normal brain (Fig. 5G, H). Indeed the normal brain at that age showed also some axons growing in the direction of the thalamus (red arrow in Fig. 5G), and these appeared to be more numerous in the *Pax6* mutant at the same age (red arrows in Fig. 5H).

We concluded that, in wild type as well as in *Pax6* mutant brains there is a number of short mammillary axons extended in the direction of the thalamus as well as axons coursing dorsal/caudal before the mth is formed at all.

### The mammillary axonal tree has three branches

The realization that there are some mammillary axons unaccounted for in the current descriptions of the mammillary axonal tree, prompted us to examine the normal development of this fiber system using *Foxb1-tau-lacZ* heterozygotes. *Foxb1* is specificallly expressed by neurons of the MBO as well as by the dorsal premammillary nucleus (DPM) [29]. The first pm axons can be seen at E10.5 growing towards the tegmentum (Fig. 6A, B) [32]. At E14.5 some axons from the pm start growing towards the tectum—they form the mtc (Fig. 6C, D). A pronounced bend in the pm is visible at E14.5 (Fig. 6C, D) and increases through E16.5 (Fig. 6E, F) and E18.5 (Fig. 6G, H). It is precisely in this bend that the mth develops. Although the first sprouts of the mth can be seen at E17.5 (not shown), its full extent however is only visible from E18.5 on (Fig. 6G, H), more than a full week later than the earliest pm axons (Fig. 6A, B). In agreement with the beta-galactosidase data and our previous results [7], at E18.5 three components of the mammillary axonal tree (mtc, mtg and mth) can be anterogradely visualized by injecting DiI tracer in the MBO (Fig. 6I). This confirms that the mammillary body generates not two but three axonal bundles (Fig. 6J, K).

### The “problem axons” in the *Pax6* mutant are probably misdirected mtc axons

**Figure 7:**
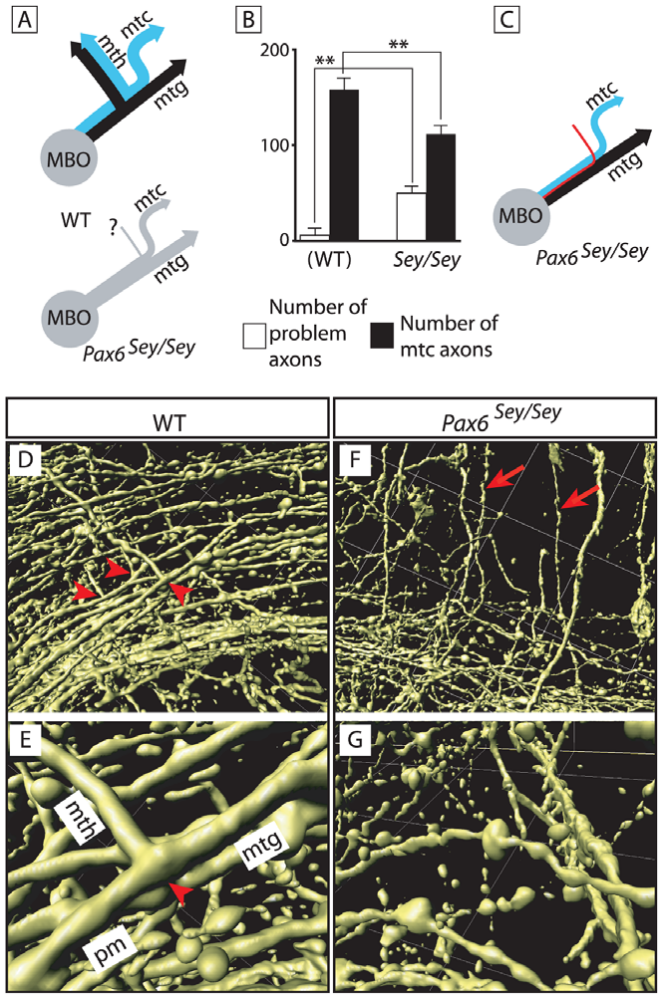
The problem axons in the *Pax6*-deficient diencephalon are mammillotectal. A) Diagram showing the component axons of the MBO in wild type (top) and *Pax6* mutant (bottom). In the wild type in blue, axons from the dorsal premammillary nucleus. In the mutant, problem axons are labeled by a question mark. B) Problem axons increase and mtc axons decrease in the *Pax6* mutant. Mean +/− SD; (**) P<0.01. C) Interpretation of the axon counting results in (B). The problem axons (red) are mtc axons initially directed dorsally and unable to turn caudally towards the tectum. D–G) 3D reconstruction of confocal images from DiI-traced pm branching point of wild type (D, E) and *Pax6*-deficient (F, G) E18.5 brains at lower (D, F) and higher (E, G) magnification. D, E) Obviously bifurcated axons can be found in the wild type branching point. F, G) Mutant axons show the characteristic beads but no branching out of them.

Our observations suggested that the problem axons seen in the *Pax6* mutant are not the product of pm branching, but simply an increased number of the mtc axons also found in normal animals. In that case, they would not be the product of a branching event but simply misdirected axons that set out in the wrong path and are unable to proceed (schematized in Fig. 7A). We reasoned that, if in the *Pax6* mutant there is an increase in the number of mtc axons inappropriately navigating towards the thalamus, then there must be a smaller number of properly oriented mtc axons. We therefore counted the mtc axons and the problem axons in *Foxb1-tau-lacZ* heterozygous and in double mutants (*Foxb1-tau-lacZ* heterozygous/*Sey* homozygous). To prevent some mtc axons from being hidden by the mth, we performed the countings at E16.5, when the mth has not yet been formed.

Our results show (Fig. 7B) that the *Foxb1-tau-lacZ* heterozygotes (i.e. normal animals) have a certain small number of problem axons [33], confirming our previous observation (Fig. 5G, red arrow). *Sey/Sey* mutants displayed significantly more problem axons than normal animals (Fig. 7B, compare white bars). Next we counted the mtc axons in the same samples and found that the *Sey/Sey* mutant had significantly less mtc axons than the normal animals (Fig. 7B, compare black bars), and that the difference in number approximately matched the difference found in problem axons. These results support the hypothesis that the problem axons are misdirected mtc axons and not the product of pm branching (schematized in Fig. 7C). Since *Pax6* is not expressed by the MBO, the effect is non cell-autonomous and caused by the scattered cells, which control navigation in this area.

### The pm does not branch in *Pax6*-deficient mutants

In order to directly confirm that there is no morphological branching of the pm axons in the *Sey/Sey* mutant, we took resource to 3-D confocal microscopy imaging of our DiI data. In the wild type E18.5 brain, at low magnification, it was possible to observe images of axonal bifurcation (arrowheads in Fig. 7D) which could be confirmed at high magnification (arrowhead in Fig. 7E).

The same technique made obvious that the axons that can be seen in *Sey/Sey* at right angles with the principal mammillary (red arrows in Fig. 7F) do not arise from bifurcations. In wild type brains, at earlier stages, the pm axons show swellings or varicosities from where the branches arise (not shown). Later, axons become thicker and the varicosities disappear (Fig. 7D, E). Interestingly, in the *Sey/Sey* mutant brain, which does not have a mammillothalamic tract, the axonal varicosities were still present at this age, but no branches where visible (Fig. 7G). We concluded that the *Pax6* mutant shows a specific non-cell autonomous defect in pm branching.

### Normal mth outgrowth in mutants with severe thalamic phenotypes and intact PTh/VTh

**Figure 8:**
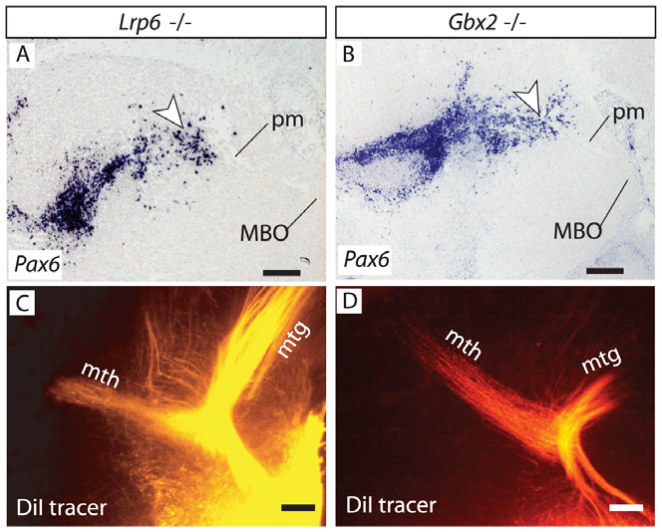
Mammillary branching is present in several thalamic mutants. A, B) *Pax6* in situ hybridization shows that PTh/VTh and branching point cells are present in the *Lrp6* mutant (A) and the *Gbx2* mutant (B) brains at E18.5. C, D) DiI tracing demonstrates presence of a mth in these mutants (C, D). Scale bars 100 micrometers.

*Pax6* is expressed in the early dorsal thalamus, target of the mth. If target attraction was essential for pm branching, differentiation defects in the *Pax6*-deficient thalamus [34] could contribute to the branching defect. If on the other hand target attraction was not essential for pm branching, mutant mouse brains showing an altered thalamus but preserving a normal PTh/VTh (together with branching point cells) should have a mth. The gene *Lrp6* encodes an important co-receptor of Wnt ligands expressed in the thalamus [35], [36]. Accordingly, the thalamus of *Lrp6* mutant mice is dramatically defective and unable to develop thalamocortical efferents [37]. Our analysis shows, however, that the *Lrp6* mutant PTh/VTh expresses *Pax6*, and *Pax6*-expressing branching point cells are present in the appropriate position around the pm (Fig. 8A). *Gbx2* is a transcription factor gene essential for thalamus differentiation, and *Gbx2* mutant mice show severely impaired thalamic development and absence of thalamocortical axons [38]–[40]. *Pax6* was expressed in the *Gbx2* mutant PTh/VTh and there were *Pax6*-expressing cells in the cell groups around the pm branching point (Fig. 8B). Consistently, DiI tracings showed that the pm branches into a mth of normal appearance in *Lrp6* mutants (Fig. 8C) and *Gbx2* mutants (Fig. 8D). Together, these results suggest that an intact thalamus is not a precondition for the initial outgrowth of mth axons for as long as the local interactions (e.g. with the *Pax6*-expressing branching point cells) are maintained.

### Axonal fasciculation and cell aggregation impaired in the *Foxb1/Pax6* double mutant

**Figure 9:**
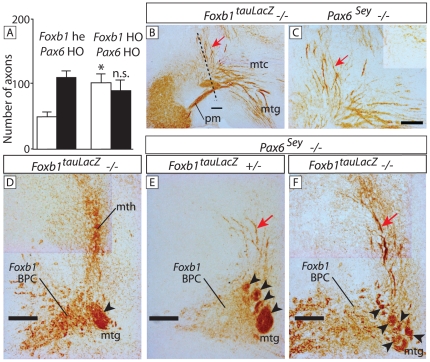
Axonal fasciculation and cell aggregation impaired in the *Foxb1/Pax6* double mutant. A) Slight increase in problem axons but no detectable change in mtc axons in the *Pax6* mutant. White column, problem axons; black column, mtc axons. Mean +/− SD; (*) P<0.05; n.s. not significant. B, C) Beta galactosidase detection on sagittal section of double *Foxb1-tauLacZ/Sey* homozygote. The problem axons (red arrow) seem more numerous as in single *Sey* homozygotes. The dotted line in (B) indicates the approximate plane of section of D, E, F. (C) shows a high magnification detail of the image in (B). D–F) Beta galactosidase detection on sections along the dotted line in (B) (left side is shown) through the branching point of E18.5 brains (genotypes as indicated). In *Foxb1* single homozygotes (D) there is a mth (branching takes place), the mtg is not subdivided into fascicles and the *Foxb1* branching point cells are tightly aggregated. In the double mutant (F), the problem axons (red arrow in E, F) are longer and more numerous, the *Foxb1*-expressing branching point cells (*Foxb1* BPC in Fig. 9D–F) are less compactly aggregated and the mtg is divided in more fascicles (arrowheads) as in the *Foxb1* heterozygote/*Sey* homozygote (E). Scale bars 100 micrometers.

*Foxb1::tau-lacZ* homozygotes show a mth navigational phenotype that has been analyzed [7]. They showed however no alteration in mtg or mtc. Double homozygous brains for *Foxb1::tau-lacZ* and *Sey*, however, showed a slight increase in the number of misguided mtc axons (former “problem axons”). This increase was statistically significant (Fig. 9A) (see also Table 1) and histologically visible (compare Fig. 5E, F with Fig. 9B, C red arrows) but not large enough to be reflected in a significant decrease of mtc axons (Fig. 9A). We then used sections along the dotted line in Fig. 9B to analyze the mtg. While in single *Foxb1* homozygotes the mtg consisted of one compact axonal bundle (arrowhead in Fig. 9D), in *Foxb1* heterozygous/*Sey* homozygous brains the mtg was subdivided in a number of bundles (arrowheads in Fig. 9E). Double homozygotes showed an mtg disgregated into numerous smaller axonal fascicles (arrowheads in Fig. 9F). The *Foxb1*-expressing branching point cells showed also progressively impaired aggregation (Fig. 9D through F).

We concluded that *Foxb1* has a role in the control of cell adhesion and axonal fasciculation. This role could be non cell-autonomous (through loss-of-function in the *Foxb1*-expressing branching point cells) or cell autonomous (since the neurons originating the mtc and mtg axons express *Foxb1*). In this way *Foxb1* cooperates with a non-cell autonomous role of *Pax6* to guarantee the appropriate anatomy of the mammillary tree.

## Discussion

How does the immediate cellular environment contribute to the formation and navigation of different fiber bundles in a complex, stereotyped axonal tree? While *in vitro* evidence suggests that the local environment could secret a variety of factors eliciting branching (see below), no example of a group of identified cells has been found which is essential for the formation of a specific axonal bundle by collateral branching in a certain system. We have identified an elaborate arrangement of specific cell populations migrating from different sources and converging around the branching point of a major forebrain axonal tract, the pm. We offer several kinds of evidence (digital reconstruction of confocal images, axon counting, analysis of mutants with differential phenotypes) pointing to an indispensable role of these cells in collateral branching and navigation. This concept complements previous reports of axonal guidance supported by surrounding cells that serve as guideposts [41].

### Several cell groups organize around the pm branching point

We show that migration from the PTh/VTh as well as from the hypothalamus results in several groups of cells arranged around the future pm branching point. Some of the *Pax6*-expressing cells could arise in a *Pax6*-expressing domain of the midbrain neuroepithelium [26]. Particularly curious is the cell group at the caudal side of the pm branching point, formed as the meeting point of *Foxb1*-expressing and *Pax6*-expressing cells originated respectively in the ventral and dorsal diencephalon. Although the diencephalon is the source of extensive non-radial migrations across dorso-ventral and rostro-caudal boundaries [16], formation of such cell groups of heterogeneous origin is not obvious from current paradigms of hypothalamic development [42]–[44]. Arranged along axonal bundles, some of the *Pax6*-expressing cells could act as guideposts for mth axons as shown for other systems [45]–[47] and the *Foxb1*-expressing cells could fulfill a similar role for pm axons.

### *Pax6*-expressing cells and collateral branching

We show 1) that the *Pax6*-positive cells that surround the pm branching point are absent in *Pax6*-deficient brains and 2) that this absence is ensued by major alterations in the axonal tree. We have investigated these alterations with a specific genetic marker of mammillary axons (the *Foxb1::tau-lacZ* allele) and digital reconstructions of confocal microscopy data to show unambiguously that the pm axons do not branch in the mutant. Previous descriptions of a number of pm collaterals in *Sey/Sey* brains [33], [48] probably result from unintentional co-labeling (DiI tracing or silver impregnation) of the mtc when attempting to label MBO projections.

Our results strongly suggest that close contact with the *Pax6*-expressing cells plays a role in fulfilling the potential of the initial branching bud. This agrees with previous work showing that direct physical contact with growth factor-soaked beads elicits branching in cultured axons [49], [50] (see [51] for a review) and that contact with nearby dendrites enhances collateral branching of cortico-spinal axons [4]. That humoral factors, including those locally secreted, can elicit axonal branching is well established (see for instance [52]) and therefore an altered *Pax6*-deficient thalamus [34], [46], [53], [54] could cause the pm branching failure through lack of target attraction as shown in other models [2], [55], [56]. Our finding that a mth is present in mutants showing severe thalamic differentiation defects while preserving *Pax6*-expressing cells around the pm branching point (Fig. 8) rather reinforces the notion that, in this model, branching and initial outgrowth depend on the local influence of a specific cell group.

### *Pax6*, *Foxb1* and adhesion

Adhesion proteins have a role in collateral branching [57] and specifically in mth development [58]. Abundant literature shows that a number of adhesion-related genes are downregulated in *Sey/Sey* brains: *cadherin 4* [45], [59], *L1cam* [60], *alpha 5 beta 1 integrin* [61], *olfactomedin 3* (*optimedin*) [62], *delta catenin* [63], *tenascin C* [64], *semaphorin-3c* and *semaphorin-a5* [65]. Intriguingly, *Pax6* seems to be involved in the pruning of inappropriate collateral branches of cortical pyramidal neurons [66]. Finally, previous analyses of *Pax6*-deficient phenotypes support a function for this gene in contact guidance of pioneer axons in the forebrain [45]–[47], [65], [67].

In contrast, *Fox* transcription factors have not been associated with adhesion gene expression [68], [69], with the possible exceptions of *Vcam1* [70] and *Cdh7* [71].

### The mtc

An interesting observation made previously by us [7] and confirmed here is the existence of the mtc. Mammillo-tectal axons homologous to our mtc have been traced in the adult rat [8]. Specific expression of *Foxb1* in the dorsal premammillary nucleus [7], [29] as well as in the MBO evidences a molecular kinship of these nuclei and supports the proposed inclusion of the dorsal premammillary in an extended definition of the MBO [8]. The mtc bundle should be included in any discussion of the formation of the mammillary axonal tree. We show that non cell-autonomous *Pax6* expression is essential for mtc navigation. Intriguingly, although the abundant *netrin 1*-expressing cells in the mammillary region do not express *Pax6*, their position around the mammillary axons is dramatically altered in *Pax6* mutant brains [33], [67]. This suggests that the *Pax6*-expressing cells of the PTh/VTh secret a not yet identified factor contributing to the appropriate positioning of the *netrin 1*-expressing cells and, through this effect, they could influence also mammillary axonal navigation indirectly.

### *Foxb1* and *Pax6* in the control of mammillary axonal organization

The double homozygotes demonstrate a *Foxb1*-regulated component in mtc and mtg navigation, probably mediated by proteins involved in fasciculation. Since the *Foxb1*-expressing branching point cells are still present in the *Foxb1*-deficient brain, and the gene is also expressed by the neurons originating the affected axons, the role of this transcription factor could be or not cell-autonomous. A non cell-autonomous role has been suggested for the mth navigational phenotype found in the *Foxb1* mutant [7].

### Conclusions

This work uncovers a series of complex cell migration events giving rise to several specific groups of cells of different origin essential for the formation and organization of an axonal crossroads linking hypothalamus, thalamus, midbrain and hindbrain. We show that many of these cells express *Pax6*, depend on expression of this gene for their origination, and are necessary for the formation of specific axonal collaterals through branching of the pm. Finally, we show cooperation of *Pax6* and *Foxb1* in mtc and mtg navigation. This work offers new insights into the development of a specific cellular environment that favors the formation and navigation of specific axonal collaterals.

## References

[1] Harris WA, Holt CE, Bonhoeffer F (1987). Retinal axons with and without their somata, growing to and arborizing in the tectum of Xenopus embryos: a time-lapse video study of single fibres in vivo.. Development.

[2] O'Leary DD, Terashima T (1988). Cortical axons branch to multiple subcortical targets by interstitial axon budding: implications for target recognition and “waiting periods”.. Neuron.

[3] O'Leary DD, Bicknese AR, De Carlos JA, Heffner CD, Koester SE (1990). Target selection by cortical axons: alternative mechanisms to establish axonal connections in the developing brain.. Cold Spring Harb Symp Quant Biol.

[4] Bastmeyer M, Daston MM, Possel H, O'Leary DD (1998). Collateral branch formation related to cellular structures in the axon tract during corticopontine target recognition.. J Comp Neurol.

[5] Vann SD, Aggleton JP (2004). The mammillary bodies: two memory systems in one?. Nat Rev Neurosci.

[6] Hayakawa T, Zyo K (1989). Retrograde double-labeling study of the mammillothalamic and the mammillotegmental projections in the rat.. J Comp Neurol.

[7] Alvarez-Bolado G, Zhou X, Voss AK, Thomas T, Gruss P (2000). Winged helix transcription factor Foxb1 is essential for access of mammillothalamic axons to the thalamus.. Development.

[8] Canteras NS, Swanson LW (1992). The dorsal premammillary nucleus: an unusual component of the mammillary body.. Proc Natl Acad Sci U S A.

[9] Mombaerts P, Wang F, Dulac C, Chao SK, Nemes A (1996). Visualizing an olfactory sensory map.. Cell.

[10] Kloetzli JM, Fontaine-Glover IA, Brown ER, Kuo M, Labosky PA (2001). The winged helix gene, Foxb1, controls development of mammary glands and regions of the CNS that regulate the milk-ejection reflex.. Genesis.

[11] Labosky PA, Winnier GE, Jetton TL, Hargett L, Ryan AK (1997). The winged helix gene, Mf3, is required for normal development of the diencephalon and midbrain, postnatal growth and the milk-ejection reflex.. Development.

[12] Wehr R, Mansouri A, de Maeyer T, Gruss P (1997). Fkh5-deficient mice show dysgenesis in the caudal midbrain and hypothalamic mammillary body.. Development.

[13] Kaestner KH, Schutz G, Monaghan AP (1996). Expression of the winged helix genes fkh-4 and fkh-5 defines domains in the central nervous system.. Mech Dev.

[14] Zhao T, Zhou X, Szabo N, Leitges M, Alvarez-Bolado G (2007). Foxb1-driven Cre expression in somites and the neuroepithelium of diencephalon, brainstem, and spinal cord.. Genesis.

[15] Soriano P (1999). Generalized lacZ expression with the ROSA26 Cre reporter strain.. Nat Genet.

[16] Zhao T, Szabo N, Ma J, Luo L, Zhou X (2008). Genetic mapping of Foxb1-cell lineage shows migration from caudal diencephalon to telencephalon and lateral hypothalamus.. Eur J Neurosci.

[17] Hill RE, Favor J, Hogan BL, Ton CC, Saunders GF (1991). Mouse small eye results from mutations in a paired-like homeobox-containing gene.. Nature.

[18] Hogan BL, Horsburgh G, Cohen J, Hetherington CM, Fisher G (1986). Small eyes (Sey): a homozygous lethal mutation on chromosome 2 which affects the differentiation of both lens and nasal placodes in the mouse.. J Embryol Exp Morphol.

[19] St-Onge L, Sosa-Pineda B, Chowdhury K, Mansouri A, Gruss P (1997). Pax6 is required for differentiation of glucagon-producing alpha-cells in mouse pancreas.. Nature.

[20] Kokubu C, Heinzmann U, Kokubu T, Sakai N, Kubota T (2004). Skeletal defects in ringelschwanz mutant mice reveal that Lrp6 is required for proper somitogenesis and osteogenesis.. Development.

[21] Simmons DM, Arriza JL, Swanson LW (1989). A complete protocol for in situ hybridization of messenger RNAs in brain and other tissues with radiolabeled single-stranded RNA probes.. J Histotechnol.

[22] Yaylaoglu MB, Titmus A, Visel A, Alvarez-Bolado G, Thaller C (2005). Comprehensive expression atlas of fibroblast growth factors and their receptors generated by a novel robotic in situ hybridization platform.. Dev Dyn.

[23] Kiecker C, Lumsden A (2004). Hedgehog signaling from the ZLI regulates diencephalic regional identity.. Nat Neurosci.

[24] Puelles L, Martínez S, Martínez-de-la-Torre M, Rubenstein JLR, Paxinos G (2004). Gene Maps and Related Histogenetic Domains in the Forebrain and Midbrain.. The Rat Nervous System.

[25] Scholpp S, Lumsden A (2010). Building a bridal chamber: development of the thalamus.. Trends Neurosci.

[26] Stoykova A, Gruss P (1994). Roles of Pax-genes in developing and adult brain as suggested by expression patterns.. J Neurosci.

[27] Stoykova A, Fritsch R, Walther C, Gruss P (1996). Forebrain patterning defects in Small eye mutant mice.. Development.

[28] Alpeeva EV, Makarenko IG (2009). Perinatal development of the mammillothalamic tract and innervation of the anterior thalamic nuclei.. Brain Res.

[29] Alvarez-Bolado G, Zhou X, Cecconi F, Gruss P (2000). Expression of Foxb1 reveals two strategies for the formation of nuclei in the developing ventral diencephalon.. Dev Neurosci.

[30] Miura H, Yanazawa M, Kato K, Kitamura K (1997). Expression of a novel aristaless related homeobox gene ‘Arx’ in the vertebrate telencephalon, diencephalon and floor plate.. Mech Dev.

[31] Jelsing J, Larsen PJ, Vrang N (2008). Identification of cannabinoid type 1 receptor expressing cocaine amphetamine-regulated transcript neurons in the rat hypothalamus and brainstem using in situ hybridization and immunohistochemistry.. Neuroscience.

[32] Easter SS, Ross LS, Frankfurter A (1993). Initial tract formation in the mouse brain.. J Neurosci.

[33] Tsuchiya R, Takahashi K, Liu FC, Takahashi H (2009). Aberrant axonal projections from mammillary bodies in Pax6 mutant mice: possible roles of Netrin-1 and Slit 2 in mammillary projections.. J Neurosci Res.

[34] Pratt T, Vitalis T, Warren N, Edgar JM, Mason JO (2000). A role for Pax6 in the normal development of dorsal thalamus and its cortical connections.. Development.

[35] Bafico A, Liu G, Yaniv A, Gazit A, Aaronson SA (2001). Novel mechanism of Wnt signalling inhibition mediated by Dickkopf-1 interaction with LRP6/Arrow.. Nat Cell Biol.

[36] Brown SD, Twells RC, Hey PJ, Cox RD, Levy ER (1998). Isolation and characterization of LRP6, a novel member of the low density lipoprotein receptor gene family.. Biochem Biophys Res Commun.

[37] Zhou CJ, Pinson KI, Pleasure SJ (2004). Severe defects in dorsal thalamic development in low-density lipoprotein receptor-related protein-6 mutants.. J Neurosci.

[38] Miyashita-Lin EM, Hevner R, Wassarman KM, Martinez S, Rubenstein JL (1999). Early neocortical regionalization in the absence of thalamic innervation.. Science.

[39] Hevner RF, Miyashita-Lin E, Rubenstein JL (2002). Cortical and thalamic axon pathfinding defects in Tbr1, Gbx2, and Pax6 mutant mice: evidence that cortical and thalamic axons interact and guide each other.. J Comp Neurol.

[40] Szabo NE, Zhao T, Zhou X, Alvarez-Bolado G (2009). The role of Sonic hedgehog of neural origin in thalamic differentiation in the mouse.. J Neurosci.

[41] Zhou L, Bar I, Achouri Y, Campbell K, De Backer O (2008). Early forebrain wiring: genetic dissection using conditional Celsr3 mutant mice.. Science.

[42] Altman J, Bayer SA (1986). The Development of the Rat Hypothalamus.. Adv Anat Embryol Cell Biol.

[43] Bayer SA, Altman J, Paxinos G (1995). Neurogenesis and Neuronal Migration.. The Rat Nervous System.

[44] Bayer SA, Altman J, Paxinos G (1995). Principles of Neurogenesis, Neuronal Migration, and Neural Circuit Formation.. The Rat Nervous System.

[45] Andrews GL, Mastick GS (2003). R-cadherin is a Pax6-regulated, growth-promoting cue for pioneer axons.. J Neurosci.

[46] Mastick GS, Davis NM, Andrew GL, Easter SS (1997). Pax-6 functions in boundary formation and axon guidance in the embryonic mouse forebrain.. Development.

[47] Nural HF, Mastick GS (2004). Pax6 guides a relay of pioneer longitudinal axons in the embryonic mouse forebrain.. J Comp Neurol.

[48] Valverde F, Garcia C, Lopez-Mascaraque L, De Carlos JA (2000). Development of the mammillothalamic tract in normal and Pax-6 mutant mice.. J Comp Neurol.

[49] Gallo G, Letourneau PC (1999). Different contributions of microtubule dynamics and transport to the growth of axons and collateral sprouts.. J Neurosci.

[50] Kalil K, Szebenyi G, Dent EW (2000). Common mechanisms underlying growth cone guidance and axon branching.. J Neurobiol.

[51] Gibson DA, Ma L (2011). Developmental regulation of axon branching in the vertebrate nervous system.. Development.

[52] Jeanneteau F, Deinhardt K, Miyoshi G, Bennett AM, Chao MV (2010). The MAP kinase phosphatase MKP-1 regulates BDNF-induced axon branching..

[53] Grindley JC, Hargett LK, Hill RE, Ross A, Hogan BL (1997). Disruption of PAX6 function in mice homozygous for the Pax6Sey-1Neu mutation produces abnormalities in the early development and regionalization of the diencephalon.. Mech Dev.

[54] Mastick GS, Andrews GL (2001). Pax6 regulates the identity of embryonic diencephalic neurons.. Mol Cell Neurosci.

[55] Bastmeyer M, O'Leary DD (1996). Dynamics of target recognition by interstitial axon branching along developing cortical axons.. J Neurosci.

[56] Sato M, Lopez-Mascaraque L, Heffner CD, O'Leary DD (1994). Action of a diffusible target-derived chemoattractant on cortical axon branch induction and directed growth.. Neuron.

[57] Daston MM, Bastmeyer M, Rutishauser U, O'Leary DD (1996). Spatially restricted increase in polysialic acid enhances corticospinal axon branching related to target recognition and innervation.. J Neurosci.

[58] Weinhold B, Seidenfaden R, Rockle I, Muhlenhoff M, Schertzinger F (2005). Genetic ablation of polysialic acid causes severe neurodevelopmental defects rescued by deletion of the neural cell adhesion molecule.. J Biol Chem.

[59] Stoykova A, Gotz M, Gruss P, Price J (1997). Pax6-dependent regulation of adhesive patterning, R-cadherin expression and boundary formation in developing forebrain.. Development.

[60] Meech R, Kallunki P, Edelman GM, Jones FS (1999). A binding site for homeodomain and Pax proteins is necessary for L1 cell adhesion molecule gene expression by Pax-6 and bone morphogenetic proteins.. Proc Natl Acad Sci U S A.

[61] Duncan MK, Kozmik Z, Cveklova K, Piatigorsky J, Cvekl A (2000). Overexpression of PAX6(5a) in lens fiber cells results in cataract and upregulation of (alpha)5(beta)1 integrin expression.. J Cell Sci.

[62] Grinchuk O, Kozmik Z, Wu X, Tomarev S (2005). The Optimedin gene is a downstream target of Pax6.. J Biol Chem.

[63] Duparc RH, Boutemmine D, Champagne MP, Tetreault N, Bernier G (2006). Pax6 is required for delta-catenin/neurojugin expression during retinal, cerebellar and cortical development in mice.. Dev Biol.

[64] von Holst A, Egbers U, Prochiantz A, Faissner A (2007). Neural stem/progenitor cells express 20 tenascin C isoforms that are differentially regulated by Pax6.. J Biol Chem.

[65] Jones L, Lopez-Bendito G, Gruss P, Stoykova A, Molnar Z (2002). Pax6 is required for the normal development of the forebrain axonal connections.. Development.

[66] Pinon MC, Tuoc TC, Ashery-Padan R, Molnar Z, Stoykova A (2008). Altered molecular regionalization and normal thalamocortical connections in cortex-specific Pax6 knock-out mice.. J Neurosci.

[67] Vitalis T, Cases O, Engelkamp D, Verney C, Price DJ (2000). Defect of tyrosine hydroxylase-immunoreactive neurons in the brains of mice lacking the transcription factor Pax6.. J Neurosci.

[68] Tuteja G, Kaestner KH (2007). SnapShot: Forkhead Transcription Factors II.. Cell.

[69] Tuteja G, Kaestner KH (2007). SnapShot: forkhead transcription factors I. Cell.

[70] Mahlapuu M, Ormestad M, Enerback S, Carlsson P (2001). The forkhead transcription factor Foxf1 is required for differentiation of extra-embryonic and lateral plate mesoderm.. Development.

[71] Dottori M, Gross MK, Labosky P, Goulding M (2001). The winged-helix transcription factor Foxd3 suppresses interneuron differentiation and promotes neural crest cell fate.. Development.

